# Development of breast cancer mortality considering the implementation of mammography screening programs – a comparison of western European countries

**DOI:** 10.1186/s12889-019-7166-6

**Published:** 2019-06-26

**Authors:** Yukio Iwamoto, Simone Kaucher, Eva Lorenz, Till Bärnighausen, Volker Winkler

**Affiliations:** 1 Institute of Global Health, Unit of Epidemiology and Biostatistics, University of Heidelberg, Im Neuenheimer Feld 324, 69120 Heidelberg, Germany; 2 Institute of Medical Biostatistics, Epidemiology and Informatics, University Medical Center, Mainz, Germany; 3 Bernhard Nocht Institute for Tropical Medicine, Research Group Infectious Disease Epidemiology, Bernhard-Nocht-Str. 74, 20359 Hamburg, Germany

**Keywords:** Breast cancer, Mortality, Mammography screening, Europe

## Abstract

**Background:**

Triggered by the successive implementation of organized mammography screening programs (MSPs) throughout western European countries over the last decades, there is an ongoing debate questioning their effectiveness. Since it is difficult to assess the effect of MSPs on a population level, we rather aim to assess the impact of the implementation itself on breast cancer mortality rates utilizing an ecological study design.

**Methods:**

We analyzed age group-specific (50–59, 60–69 and 70–79 years) female breast cancer mortality rates in 14 western European countries between 1980 and 2017 using Joinpoint regression, interrupted time series (ITS) regression and multivariable Poisson regression.

**Results:**

The Joinpoint analysis demonstrated decreasing trends resulting in annual percentage changes ranging from − 1.5% to − 5.4% (50–59), − 0.2% to − 8.1% (60–69) and 0% to − 7.1% (70–79) depending on the country within 3 years after MSP implementation. The ITS analysis results in highly significant interaction terms (calendar year * binary MSP indicator) for all age groups. The multivariable regression using “calendar year”, “year of MSP implementation” and “years with MSP” as independent variables yielded a significant yearly decrease for “years with MSP” ranging from 0.9 to 1.2%.

**Conclusions:**

The results of this study suggest a positive association between the implementation of MSPs and the (accelerated) reduction of breast cancer mortality rates. Measuring and quantifying the isolated effect of MSPs on a population level will require additional studies using individual data.

## Background

In most western European countries, breast cancer is the leading malignant neoplasm among women [1]. Its disease burden has been recognized as a major public health issue across many countries.

In the early 1980s, a controlled trial was conducted in two counties of Sweden to examine the effect of an organized breast cancer screening program which resulted in an estimated reduction of 30% in long-term breast cancer mortality [2]. Since then, additional randomized controlled trials assessing the effectiveness and other important aspects around mammography screening programs (MSPs) were performed in other Swedish counties as well as European countries [3–6]. Promising study results and the decision of the “Committee of Cancer Experts of the European Community” in 1986 led to a roll-out of organized MSPs in many European countries [7, 8]. These MSPs have been executing their planning, implementation, and quality assurance according to the “European guidelines for quality assurance in breast cancer screening and diagnosis”, which were introduced in 1993 from the “European reference organization for quality assured breast screening and diagnostic services” (EUREF) and are now available in its 4th version from 2006 [9]. These guidelines include a quality-controlled x-ray based diagnostic approach of a biennial screening program offered to women in the age range between 50 and 69 years, by trained professionals who should work together in certified breast centers during the entire process of invitation, diagnostics, therapy and follow-up of participants. The EUREF defined about 40 performance indicators with acceptable and desirable levels, which are supposed to be monitored continuously. However, none of the European MSPs achieved these recommendations for all performance indicators at an acceptable level [7]. Especially the acceptable participation of at least 70% is not reached by all countries (see Table 1).

**Table 1:** MSPs in western European countries with more than 4.5 million inhabitants [7, 8, 10–15]

Country	Implementation Period^a^	Age range of women eligible for screening	Participation rate	Years for analysis
Austria^b^	2014	50–69	–	2004–2017
Belgium^c^	2001	50–69	2005: 38% [7]	1991–2015
Denmark^d^	2007–2010	50–69	2010: 73% [12]	1997–2015
Finland^e^	1987	50–69	2010: 85% [12]	1980–2015
France^f^	1989–2004	50–74	2010: 52% [12]	1980–2015
Germany	2005–2009	50–69	2014: 54% [14]	1995–2015
Italy	2002–2007	50–69	2010: 61% [12]	1992–2015
Netherlands^g^	1988–1997	50–75	2010: 81% [12]	1980–2016
Norway	1996–2004	50–69	2010: 76% [12]	1986–2016
Portugal^h^	1990–1999	45–69	2010: 63/58% [12]	1989–2016
Spain^i^	1990–2003	50–69	2015: 75% [13]	1980–2016
Sweden^j^	1986–1996	40–74	2010: 70% [12]	1980–2016
Switzerland^k^	1999–2004	50–70	2012: 46% [16]	1989–2015
United Kingdom^l^	1988–1995	50–70	2010: 73% [12]	1980–2015

^a^The implementation period is defined as the year when the roll-out of a national MSP began until the year in which a 100% geographical national coverage was achieved. The degree of completeness, measurable e.g. by % coverage by invitation, as well as pace and strategy of roll-out varies between countries

^b^In Austria, an opportunistic screening without an invitation mechanism or dedicated mammography screening centers was introduced in 1974, but an organized MSP was only introduced in 2014

^c^In Belgium, the participation rate for 2005 is just for the province Flanders

^d^In Denmark, a population-based screening program started in 1991 in Copenhagen and in 1993 in the region of Funen. In 2007, nationwide MSP was rolled out

^e^In Finland, in some regions, the upper limit of invitation is 59, while in others 69

^f^In France, partial regional screening was introduced in 1989, followed by a partial national screening covering some parts of France from 1994. The MSP was initially planned for women aged 50–69 and the age-range was then extended to 50–74 [17]

^g^In the Netherlands, an MSP for women aged 50–69 was implemented from 1987 to 1997 and extended to age 74 from 1998 to 2001 [18]

^h^In Portugal, an MSP was launched in 1990 in the northern Central Region and in 1997 in the southern Alentejo Region. The participation rates are therefore mentioned separately for these regions (63% in Central, 58% in Alentejo)

^i^In Spain, the model of MSPs varies throughout the different states regarding screened age groups and initiation years. In most parts of the country, an organized MSP was introduced during 1990–2003 targeting 50–69-year-old women. Some parts start screening already at age 45, other parts stop screening at 65

^j^In Sweden, pilot projects started in 1982 and screening was recommended for women aged 50–69. Later the screened age range was extended

^k^In Switzerland, the geographical coverage for organized MSPs in 2012 was 37%, and was still at 56% in 2015 [16]. The coverage by invitation within the target population, however, was at 93% (2012) and 99% (2015) with participation rates of 46% (2012) and 41% (2015) respectively

^l^In the United Kingdom, the screened age group was 50–64, from 1988 to 2001 [8]

MSPs are not the only preventive measure conducted to reduce breast cancer mortality. The so-called “grey” or opportunistic screening is a non-organized mammography screening performed by any specialist upon recommendation to or request of the patient. In most of the countries considered in this study, the opportunistic screening could be observed before the implementation and in coexistence with systematic screening. The significant difference to organized programs is the lack of a systematic invitation process, the absence of quality management instances and the fact that mammograms may be taken and evaluated in clinics which are not certified breast centers [19]. Most importantly there is no central documentation of the execution and the evaluation of the measure.

Across Europe, a debate emerged on the effectiveness of MSPs with regard to their ultimate goal of reducing breast cancer mortality on the population level [20, 21]. Decreasing breast cancer mortality due to advances in diagnostics (such as the evolution from film to digital imaging systems or improvements in evaluation skills for mammography among radiologists) and treatment but also opportunistic screening and low MSP participation makes it difficult to analyze the isolated effect of a population-wide MSP on breast cancer mortality [7, 19, 22].

Additional contributing factors such as attention or popularity in media, the public discussion of such an extensive health policy and consequences taken by individuals through the awareness are too heterogeneous between the different populations and are therefore analyzed as the aggregate effect of implementation. Thus, we chose an ecological study approach to compare the developments of breast cancer mortality across western European countries. Therefore, our study focuses on the effect through the implementation of the MSP policy, rather than the isolated effect of the measure itself.

## Methods

First, we descriptively compared time trends in breast cancer mortality rates across all 14 western European countries with more than 4.5 million inhabitants in relation to the years of MSP implementation applying Joinpoint regression. Second, we used interrupted time series (ITS) to quantify the time trend change after MSP implementation. Third, we utilized years of MSP implementation of each country to estimate an independent effect through MSP implementation on breast cancer mortality trends using Poisson regression.

### Data

We extracted data on the number of female deaths from breast cancer from the World Health Organization (WHO) mortality database and respective mid-year population figures from the WHO population database for the 14 western European countries from 1980 onwards (Database from December 2018) [23]. The data comprised population figures and deaths registered in national vital registration systems, with underlying causes of death as coded by the responsible national authority. These data are official national statistics and have been transferred to the WHO by the competent authorities of the countries concerned. The International Statistical Classification of Diseases and Related Health Problems (ICD) codes for breast cancer were 174 (ICD-8), 174 (ICD-9), and C50 (ICD-10).

### Statistical analysis

We calculated yearly age-specific breast cancer mortality rates for the following 10-year age groups: 50–59, 60–69, 70–79 and analyzed yearly time trends for each country separately using Joinpoint regression [24]. Joinpoint regression identifies whether there are statistically significant differences in time trends and allows to calculate annual percentage changes (APC) for periods with linear trends on a log scale [24]. In all Joinpoint analyses, log transformation and a variance estimation based on the Poisson distribution was used. In this descriptive part, the time-period for analysis was set to 10 years prior and to maximum 31 years post MSP implementation.

Joinpoint analyses were performed for each country separately while ITS was performed on the aggregated data from all 14 countries together for the time frame from 1980 to 2017 and for the 3 ten-year age groups separately. ITS is a common analysis to evaluate the effectiveness of population-based interventions retrospectively [25]. We modeled the log of the observed number of deaths using the log of the population figures as the offset and “calendar year” (coded as “year–1980”) and the interaction term between “calendar year” and a binary variable indicating “availability of MSP” in the respective year and country as given in Table 1.

Multivariable Poisson regression models were used to further investigate the association between “calendar year”, “year of MSP implementation”, “years with MSP” and breast cancer mortality rates. The “year of MSP implementation” was set to the year when MSP was first publicly available (see Table 1) and thus coded as “year of interest–1980”. “Years with MSP” is an additionally constructed variable, which counts the years since MSP implementation with values ranging from 0 to 31. For each 10-year age group, we modeled the log of the observed number of deaths using “calendar year”, “year of MSP implementation” and “years with MSP” as independent variables and the log of the population size as the offset. Statistical analyses were performed using Joinpoint Regression software (https://surveillance.cancer.gov/joinpoint/) and Stata SE Version 15.

## Results

### Development of breast cancer mortality

**Fig. 1:**
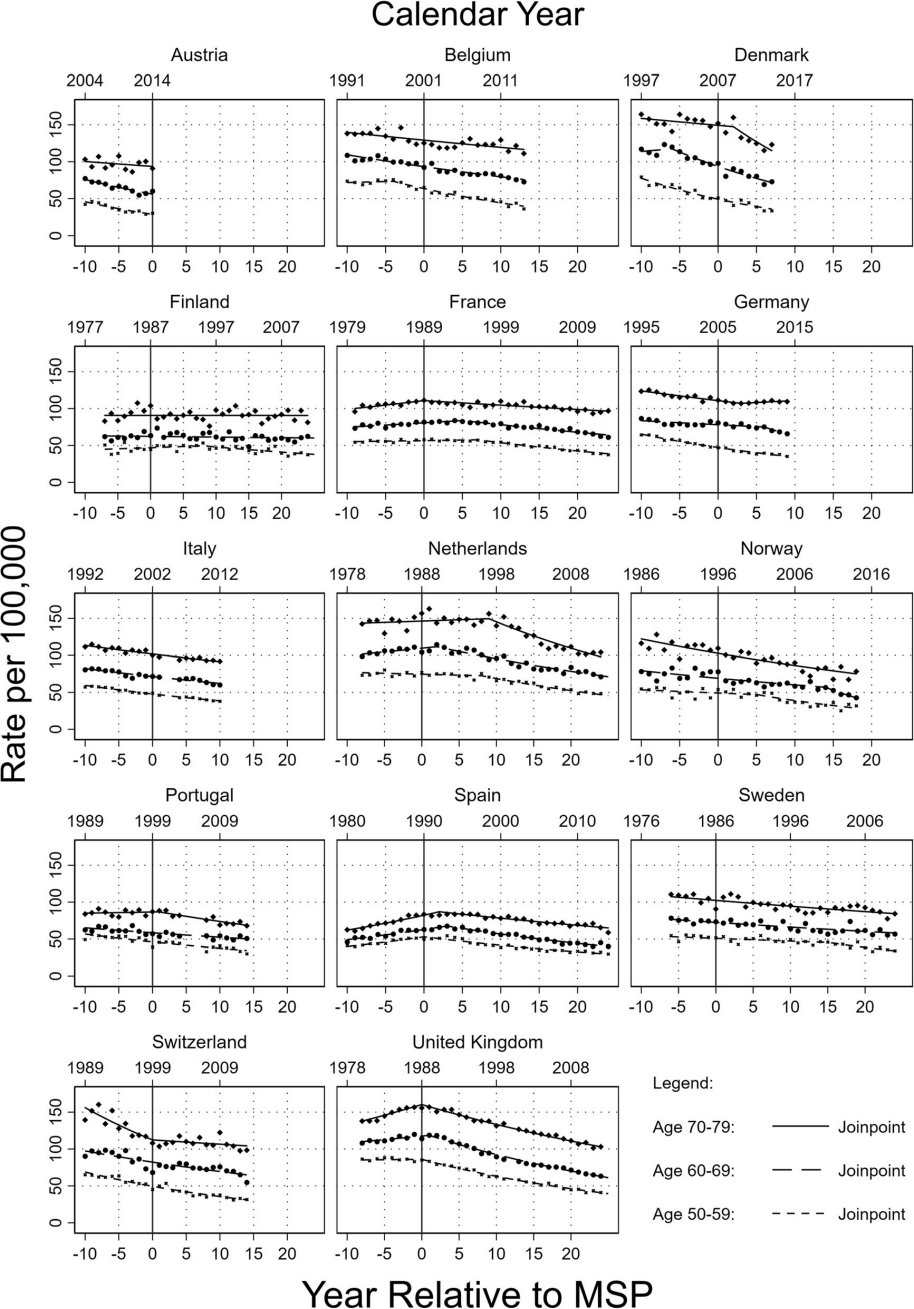
Joinpoint analysis of breast cancer-related mortality rates since 1980 in 14 western European countries

Figure 1 shows trends of breast cancer-related mortality rates (per 100,000) by country during 1980–2017 based on data from 14 western European countries. To improve comparability of results, the time frame of the Joinpoint analysis was limited to 10 years before the MSP implementations until the most recent year available in the WHO dataset from December 2018. The most recent year available varies from 2015 to 2017 depending on the country. Analysis were performed using Joinpoint regression.

Trends of breast cancer-related mortality rates are shown in Fig. 1. Overall, decreasing trends in breast cancer mortality can be observed throughout all analyzed countries, ranging from − 1.5% to − 5.4% (50–59), − 0.2% to − 8.1% (60–69) and 0% to − 7.1% (70–79) depending on the country within 3 years after MSP implementation.

In the Netherlands, women aged 50–59 years already had a declining trend of breast cancer mortality (APC: − 0.4%) before the MSP initiation in the period 1980 to 1996. However, the reduction accelerated from 1996 onwards (APC: − 2.6%). Among 60–69-year-old women, mortality rates changed from a slight increasing trend (APC: 0.9%) between 1980 and 1990 to decreasing rates from 1990 onwards (APC: − 2%). In the 70–79-year-old group, an effect of decreasing mortality rates was observed from 1997 onwards (APC < 1997: 0.3%; ≥1997: − 2.9%). In many other countries such as Spain, Portugal or France, similar patterns were observed.

In Denmark, where a nationwide MSP was only implemented in 2007, a regional MSP in densely populated areas like Copenhagen and Funen had already existed since the early 1990s [26]. The age group of 50–59–year-old women showed a constant downward trend for the entire observation period (APC: − 4.5%). Whereas mortality among 60–69-year-old women was slightly increasing before 2001 (APC: 1%), it decreased considerably thereafter (APC: − 3.9%). In the screening ineligible age group of 70–79-year-old women, breast cancer mortality was somewhat decreasing until 2007 (APC: − 0.4%) and then the trend further accelerated with the introduction of the nationwide MSP (APC: − 3.1%). In the United Kingdom, all three age groups showed a significant change of their mortality trends close to the MSP initiation in 1988 (APC 50–59 < 1988: 0.1%, ≥1988: -3.1%; APC 60–69 < 1990: 0.8%, ≥1990: -3.4%; APC 70–79 < 1988: 1.9%, ≥1988: − 1.8%).

In Finland an MSP was implemented in 1987, but in some regions, it was only offered up to age 59 and in other regions up to age 69. While for the age group of 50–59-year-old women there was a trend change in 1995 (APC < 1995: 0.7%, ≥1995: − 1.6%), the slightly decreasing trend remained constant for 60–69-year-old women (APC − 0.2%). For women aged 70–79, mortality remained constant over, showing no significant APC change in the 39 years analyzed.

In Germany, an MSP was implemented in 2005. For women aged 50–59 years, a constant decrease with an APC of − 3.1% was observed since 1994. Among 60–69-year-old women a stronger reduction was observed since 2008 (APC < 2008: − 0.1%, ≥2008: − 3%). For women aged 70–79 years, mortality slightly increased since 2008 (APC < 2008: − 1.2%, ≥2008 0.7%). In contrast, in Norway the screened age group of 50–59-year-old women showed an acceleration in the decreasing trend 4 years after the implementation of an MSP (APC < 2000: − 0.7%, ≥2000–3.5%), while the other age groups had a declining trend with no APC changes in close proximity to the implementation of an MSP.

In Italy and Sweden, a nationwide MSP implementation took a long time and varied strongly depending on the region of these countries. We observed a general decreasing trend in breast cancer mortality in these countries across all age groups, irrespective of the MSP implementation. For Belgium and Switzerland, we also observed general declining breast cancer mortality trends over time, without an accelerated reduction after MSP implementation. In Austria, an MSP was implemented in 2014; thus, it is too early to see possible changes in its decreasing trends in breast cancer mortality due to the implementation of its MSP.

### Interrupted time series regression

The results of the ITS analyses are shown in Table 2 and are based on the combined data from all 14 countries. All three age-specific models resulted in decreasing calendar year effects with yearly rate ratios (RR) of 0.987, 0.992 and 0.998 for the age groups 50–59, 60–69, 70–79, respectively. For the oldest age group, the effect was not significant. Further, all models estimated highly significant interaction terms indicating an additional decrease in breast cancer mortality with yearly RR ranging from 0.996 to 0.997.

**Table 2:** Interrupted time series regression based on combined data from all 14 western European countries

	Age 50–59		Age 60–69		Age 70–79	
	Estimate	*p*-value	Estimate	*p*-value	Estimate	*p*-value
Constant	−7.266	< 0.001	−6.988	< 0.001	−6.737	< 0.001
Calendar year (year-1980)	−0.013	< 0.001	− 0.008	0.004	− 0.002	0.190
Interaction term (Availability of MSP × Calendar year)	− 0.004	< 0.001	− 0.003	< 0.001	−0.004	< 0.001

Results of the interrupted time series regression on the association between independent variables calendar year and an interaction term for calendar year and the availability of MSP and breast cancer mortality. The analysis was performed based on combined data from all 14 western European countries considered in this study. Results are presented for the three defined age groups (50–59, 60–69, 70–79) separately.

### Multivariable Poisson regression

Results of the multivariable Poisson regression analysis are presented in Table 3 and are based on the combined data from all 14 countries. The independent effects of variables “calendar year”, “year of MSP implementation” and “years with MSP” on breast cancer mortality were also estimated in three age-specific models. In each model, the estimates that were highly significant with *p* < 0.001 are shown in bold. For the “calendar year” effect, the estimated yearly RR for the age group of 50–59 was 0.987 [95% confidence interval (CI), 0.984; 0.990], 0.996 [95% CI, 0.992; 0.999] for 60–69 and 0.992 [95% CI, 0.995; 0.999] for 70–79. The “year of MSP implementation” was estimated with an RR of 0.995 [95% CI, 0.992; 0.998] for the youngest group, 0.996 [95% CI, 0.992; 0.999] for the 60–69 age group and 0.998 [95% CI, 0.994; 0.999] for the oldest age group. “years with MSP” is in the focus of interest and results in RR estimates of 0.991 [95% CI, 0.987; 0.996], 0.988 [95% CI, 0.987; 0.993] and 0.991 [95% CI, 0.986; 0.996] for the respective age groups.

**Table 3:** Multivariable Poisson regression with independent variables: “calendar year”, “year of MSP implementation”, “years with MSP”

	Age 50–59		Age 60–69		Age 70–79	
	Estimate	95% CI	Estimate	95% CI	Estimate	95% CI
Constant	**−7.196**	−7.240 − − 7.152	**−6.950**	− 6.997 − − 6.904	**−6.703**	− 6.715 − − 6.654
Calendar year (year-1980)	**−0.013**	− 0.016 − − 0.010	**−0.004**	− 0.008 − − 0.001	−0.002	− 0.005 – 0.001
Year of MSP implementation (year-1980)	**−0.005**	− 0.008 − − 0.002	**−0.004**	− 0.008 − − 0.001	−0.002	− 0.006 − 0.001
Years with MSP (0–31)	**− 0.009**	−0.013 − − 0.004	**−0.012**	− 0.017 − − 0.007	**−0.009**	− 0.014 − − 0.004

Results of multivariable Poisson regression of the association between “calendar year”, “year of MSP implementation” and “years with MSP” and breast cancer mortality in three different age groups (50–59, 60–69, 70–79). The analysis was performed based on combined data from all 14 western European countries considered in this study. Effect estimates with *p*-values< 0.001 are indicated in bold.

## Discussion

Overall, the development of breast cancer mortality trends across 14 western European countries supports previous studies on the effectiveness of the implementation of MSPs on the reduction of breast cancer mortality. Results from descriptive as well as regression analyses support an overall positive effect of MSP implementation on declining breast cancer mortality by suggesting an accelerated decline after MSP implementation.

However, due to the complexity of the topic and the nature of this study, the outcome has to be interpreted with caution and the following limitations have to be considered. The time frame of the Joinpoint analysis was limited to 10 years before the MSP implementations for each country, to improve comparability of the results as demonstrated in Fig. 1. The exclusion of data may lead to the exclusion of certain trend changes. However, in a sensitivity analysis (results not shown) including the complete data from 1980 onwards for all countries, the results remained mainly unchanged.

The age group of 70–79 old women does not participate directly in the MSP, but following the implementation of an MSP, every year, more women in this group were eligible for the mammography earlier in their life. Therefore, an even more delayed effect might be expected in this age group as observed in countries such as Denmark or the Netherlands. Overall, Joinpoint regression results are in line with findings from previous studies examining time trends in breast cancer mortality [27, 28]. Previous studies from Italy, Sweden and Norway reported a positive association between MSP implementation and breast cancer mortality [29–31]. These studies used mortality data on a regional level and were able to define “year of MSP implementation” more accurately than we did, which is particularly important for countries like Italy, Sweden and Norway, since the MSP implementation period was rather long in these countries and regional heterogeneity regarding the MSP implementation was observed. Therefore, the country-specific association between MSP implementation and breast cancer mortality might be somewhat blurred in our results for these countries.

Regarding the regression analyses, we want to clearly state that we did not consider a time lag between MSP implementation and mortality reduction. However, we consider this a minor problem and we think any consideration of a time lag would be artificial since this study looked at the population-wide effect of MSP implementation which includes effects due to the healthcare policy e.g. raised awareness in the population even before the implementation. Furthermore, the Joinpoint analysis suggested that an effect may already be seen in most of the countries shortly after MSP initiation. Similar breast cancer mortality trend changes soon after MSP implementation were already observed in several studies in the Netherlands [18, 32]. The authors argue that the expectation for the effect of screening revealing to its fullest after 3 to 5 years is based on randomized controlled trials from the 1980s with rather small study populations. The effect on large country-wide populations with many more cases might be underestimated due to the increased survival of women with metastasized breast cancer who, without screening, would not have been diagnosed and treated. They state the decline to be a combined effect of the increased use of adjuvant therapy and the implementation of an MSP and that it is common to see a first change in trend before the full effect of screening can be observed.

With regard to the multivariable regression, it is essential to discuss each of the incorporated variables thoroughly and be aware of their limitations. The “calendar year” effect should mainly reflect components such as the improvement of medical treatment and greater breast cancer awareness over time. In the 1980s, tamoxifen and chemotherapy were introduced, followed by the sentinel-lymph-node-biopsy in the 1990s. In the beginning of the 2000s, the positive association between hormone replacement therapy and breast cancer incidence and mortality increase was revealed in several studies and led to a far more cautious use of these ever since [33]. Another considerable advance in breast cancer treatment was the introduction of monoclonal antibody therapy in the mid-2000s against HER2+ breast cancer, which is a highly lethal type of breast cancer and contributes to 20–30% of all breast cancers [34]. Today, the therapy with antibodies like trastuzumab is standard of care for these cancers, and its introduction had an indisputable positive effect on the overall survival of patients with a prolongation of survival of more than 4.5 years [34]. The continuous improvements, especially in the sector of chemotherapy, also contributed to regular recurrence rate and subsequent mortality rate reduction [35]. Any innovative therapy is introduced successively into healthcare systems, usually starting with a small group of patients in urban areas around university hospitals followed by a slow spill over to the rest of the population. Therefore, it is difficult to isolate and consider the mentioned effects in the analysis without detailed country-specific data, which is not available for this large time frame and populations. Yet, a recent study has shown that patients participating in MSPs benefit even more from advanced therapeutic options available and advances in treatment options should therefore not be seen as an argument against but rather as a further benefit for the effectiveness of MSPs [36].

“Year of MSP implementation” tries to address continuous advances in technological diagnostics and improvements of MSP programs through quality management measures available at the initiating point in time. However, exact years of implementation are not available since implementation usually took several years. Regarding opportunistic screening, it is very challenging to quantify the scale and impact of it on a population level, and there have only been few studies comparing organized programs to opportunistic breast cancer screenings. Some demonstrated the advantages of higher sensitivities and specificities of MSPs while others stated no difference in effectiveness [19, 37]. In most of the countries, opportunistic screening is not being reimbursed by public health insurances. The implementation of MSPs and the presence of the topic through awareness campaigns in media and society could have led to an increase in opportunistic screening activity, which in the end contributed to the decrease in mortality [38].

“Years with MSP” aims to assess the independent time effect on mortality since MSP implementation.

We were not able to account for a possible non-linear association between MSP implementation and breast cancer mortality in our regression models. Modeling based on country-level aggregated data reduces the absolute sample size considerably and does not allow to adequately model more complex functional forms in order to provide estimates of satisfying precision. Furthermore, we want to explicitly state, that the time variables are partially correlated, which may yield biased estimates.

In summary, we acknowledge the limitations of the regression analyses and especially the magnitude of the modeled effects should be interpreted with caution, but results support and supplement the descriptive Joinpoint analysis.

Finally, this study does not consider in its analysis nor can give quantitative answers to some of the remaining controversial aspects discussed between supporters and critics of MSPs, including the ones that do not directly affect mortality-based effectiveness measurement of MSPs. These aspects include cost-benefit ratios, radiation-induced cancer, balance between mortality reduction vs. over-diagnosis, optimum screening age range, alternative imaging or even screening techniques and many more. Furthermore, we did not use incidence-based mortality rates even though this may have shown a stronger decline since in the early years, most deaths have already been diagnosed before MSP implementation. However, this data is not directly available for all countries. Additionally, our study did not assess the isolated effect of the MSP itself, but rather the aggregated effect of MSP implementations, which also has an effect on women who were not yet invited to the MSP. MSPs are still the only scientifically proven method for an effective population-wide early detection of breast cancer in the age range from 50 to 69 so far. Alternative imaging techniques such as sonography or MRI can certainly be helpful adjunctive screening tools for specific patient groups [39]. Novel epigenetic-based screening approaches are constantly being discussed as potential successors of MSPs, but are still only in trial stages and have yet to be proven to be reliable and feasible on a population-wide scale and especially in the age range of patients targeted by MSPs [40].

Other studies such as an evaluation of various potential influencing factors on breast cancer mortality reduction including MSPs in Switzerland using Bayesian hierarchical spatiotemporal models did not show a direct link between screening and breast cancer mortality reduction [41]. In contrast, the International Agency for Research on Cancer has published an extensive review in 2016 focusing on all the controversial aspects mentioned above. These experts from more than 16 countries concluded and reaffirmed that women attending the MSP according to the EUREF guidelines would benefit with a mortality reduction of around 40% [42, 43].

## Conclusions

Driven by the controversial discussion on MSP effectiveness and risk-benefit ratios across countries, we analyzed the overall trend across 14 western European countries over a time frame of 38 years. Our multinational ecologic study approach showed that the policy of implementing MSPs had a noticeable impact contributing to the reduction of breast cancer mortality, and further supports past studies demonstrating the effectiveness of MSPs [27, 36, 44]. However, the specific extent to which MSPs themselves contribute to reducing breast cancer mortality remains challenging to be quantified and should be an objective of further research with detailed individual data.

## Data Availability

The dataset analyzed during the current study is freely available in the WHO Mortality Database (from December 2018): http://www.who.int/healthinfo/statistics/mortality_rawdata/en/

## Abbreviations

APC: Annual percentage changes
CI: Confidence interval
EUREF: European reference organization for quality assured breast screening and diagnostic services
ICD: International Statistical Classification of Diseases and Related Health Problems
ITS: Interrupted time series
MSP: Mammography screening program
RR: Rate ratios
WHO: World Health Organization
